# *Pinus densiflora* needle supercritical fluid extract suppresses the expression of pro-inflammatory mediators iNOS, IL-6 and IL-1β, and activation of inflammatory STAT1 and STAT3 signaling proteins in bacterial lipopolysaccharide-challenged murine macrophages

**DOI:** 10.1186/s40199-017-0184-y

**Published:** 2017-08-04

**Authors:** Thamizhiniyan Venkatesan, Young-Woong Choi, Jennifer Lee, Young-Kyoon Kim

**Affiliations:** Department of Forest Products and Biotechnology, College of Forest Science, Kookmin University, 861–1 Chongnung-dong, Songbuk-gu, Seoul, 136-702 South Korea

**Keywords:** Pine needle, *Pinus densiflora*, Supercritical fluid extract, RAW 264.7 murine macrophages

## Abstract

**Background:**

Regulation of a persistently-activated inflammatory response in macrophages is an important target for treatment of various chronic diseases. Pine needle extracts are well known to have potent immunomodulatory effects. The current study was designed to evaluate the effects of *Pinus densiflora* needle supercritical fluid extract (PDN-SCFE) on bacterial lipopolysaccharide (LPS)-induced inflammatory response in RAW 264.7 murine macrophages.

**Methods:**

Cytotoxic effect of PDN-SCFE was determined by the 3-(4,5-dimethylthiazol-2-yl)-2,5-diphenyltetrazolium bromide (MTT) assay. The levels of nitric oxide (NO) and the corresponding enzyme, inducible nitric oxide synthase (iNOS), were quantified by Griess and immunoblotting methods, respectively. The levels of cytokines were quantified using commercial ELISA kits. Quantitative real-time PCR (qRT-PCR) analysis was performed to assess the mRNA expression of iNOS and cytokines. To elucidate the mechanism of action, the involvement of nuclear transcription factor-kappa B (NFκB), mitogen activated protein kinases (MAPKs) and Janus kinase-signal transducers and activators of transcription (JAK-STAT) pathways were examined by an immunoblotting method. In addition, the cellular localization of NFκB was analyzed by immunofluorescence staining.

**Results:**

MTT assay results indicated that PDN-SCFE is non-toxic to RAW 264.7 cells up to a maximum assayed concentration of 40 μg/mL. The PDN-SCFE exhibited a concentration-dependent inhibitory effect on LPS-induced NO production by down regulating the expression of iNOS. In addition, the extract suppressed the LPS-induced expression of interleukin-6 (IL-6) and interleukin-1β (IL-1β) but not tumour necrosis factor-α (TNFα). Mechanistic studies revealed that PDN-SCFE does not influence the NFκB and MAPK pathways. However, it showed a significant inhibitory effect on LPS-induced activation of STAT1 and STAT3 proteins in macrophages.

**Conclusion:**

The present findings revealed that the anti-inflammatory activity of PDN-SCFE in LPS-challenged RAW 264.7 macrophages is probably caused by the suppression of the JAK-STAT signaling pathway.

**Graphical Abstract:**

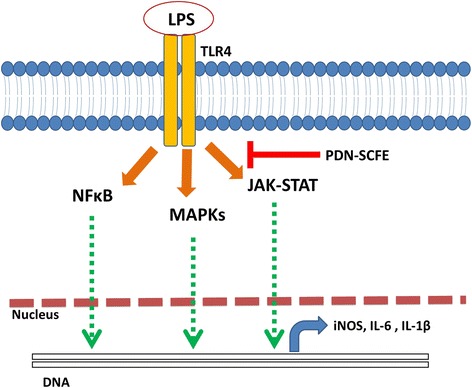

## Background

Inflammation is a complex host immunological defense reaction that generally occurs in response to invading foreign pathogens and systemic injuries. This process is usually accompanied by the activation of acute inflammatory cells followed by the synthesis and secretion of various inflammatory mediators by activated cells. The primary function of these mediators is to destroy or eliminate a foreign pathogen and also to restore tissue homeostasis [1]. However, excessive and prolonged activation of inflammatory cells and production of inflammatory mediators can promote the progression of severe tissue damage [2]. Many abnormal disease conditions such as atherosclerosis, arthritis, asthma, neurological disorders, diabetes, and cancer are associated with persistently-increased, inflammatory activity [3–7]. Thus, the suppression of chronic inflammatory reactions is an important target for the treatment of such inflammation-related diseases.

Among the various types of cells, macrophages are an important component of the mammalian immune system [8]. Macrophages are often considered to be primary, acute inflammatory cells as they provide an immediate response to the inflammatory stimuli. Further, it is well known that they largely contribute to the pathogenesis of inflammatory disease complications by producing substantial amounts of pro-inflammatory mediators, such as nitric oxide (NO), Prostaglandin E2 (PGE2), tumour necrosis factor-α (TNFα), interleukin-6 (IL-6) and interleukin-1β (IL-1β) [9]. Thus, macrophages are utilized as primary target cells for the control of the pathogenesis and complications of various inflammatory diseases. Lipopolysaccharide (LPS), a major component of the cell wall of gram-negative bacteria, is one of the most effective activators of macrophages. LPS primarily induces an inflammatory response in macrophages by binding with the membrane surface Toll-like receptor 4 (TLR4) protein. Upon stimulation by LPS, TLR4 activates its downstream nuclear transcription factor-kappa B (NFκB), the mitogen activated protein kinases (MAPKs), Janus kinase-signal transducers and activators of transcription (JAK-STAT) and various other intracellular signaling pathways to up-regulate the synthesis and secretion of inflammatory factors [9–12]. Thus, LPS-stimulated macrophages are generally utilized as an in-vitro model system for the development of anti-inflammatory drugs.

Pine trees are the most common coniferous evergreen trees comprised of around a hundred species worldwide. Several existing studies have shown that pine needle extracts possess various pharmacological properties including anti-inflammatory effects [13–16]. For instance, the needle extract of *Pinus densiflora,* one of the predominant pine species in Korea, possesses a significant inhibitory effect on NO production in LPS and interferon-γ-challenged microglia, resident monocyte-lineage cells in brain tissues [17]. In the present study, we found that the supercritical fluid extract from needle of the same pine species suppressed the inflammatory response by decreasing the production of NO as well as cytokines IL-6 and IL-1β in LPS-challenged RAW 264.7 murine macrophages.

## Methods

### Cell line, antibodies and reagents

RAW 264.7 murine macrophage cells (KCLB No. 40071) were obtained from the Korea Cell Line Bank, Seoul, Korea. Dulbecco’s modified Eagle’s medium (DMEM), antibiotics and heat-inactivated fetal bovine serum (HI-FBS) were purchased from Life Technology (Rockville, USA). Lipopolysaccharide (*E. coli*, Serotype 0111:B4), and MTT (3-(4,5-Dimethylthiazol-2-yl)-2,5-Diphenyltetrazolium Bromide) were obtained from Sigma-Aldrich (St. Louis, MO, USA). ELISA kits for TNFα, IL-6 and IL-1β were purchased from R&D Systems (Minneapolis, MN, USA). Primary antibodies to iNOS, β-actin, NFκB, and Lamin B, and Horseradish peroxidase (HRP)-conjugated secondary antibodies were bought from Santa Cruz Biotechnology, Inc. (Dallas, TX, USA). P38, phospho-P38, JNK, phospho-JNK, ERK, phospho-ERK, STAT3, phospho-STAT3 (Tyr705), STAT1, and phospho-STAT1 (Tyr701) antibodies were procured from Cell Signaling Technology (Beverly, MA, USA). Goat anti-Rabbit IgG H&L (Alexa Fluor® 488) was obtained from Abcam (Cambridge, MA).

### Plant material and supercritical fluid extraction

*Pinus densiflora* needle was collected from the local city, Seoul, Republic of Korea. The plant was identified and authenticated by the corresponding author Professor Young-Kyoon Kim. A voucher specimen (PDN-016) was placed in the herbarium of the College of Forest Science, Kookmin University, Korea. The system PHOS-NTECH, 5 L SC-CO_2_ was used to prepare supercritical fluid extract according to the manufacturer’s instructions. Briefly, 1.0 kg of dried, minced *Pinus densiflora* needle was subjected to extraction at 300 bar pressure and 60 °C temperature, for 2 h. The flow rate of carbon dioxide and ethanol per minute was adjusted to 140 mL and 10 mL, respectively. The pressure and temperature within the separating vessel were maintained at 50 bar and 25 °C, respectively. Extracts were concentrated under reduced pressure at 40 °C, using a rotary evaporator (yield, 6.2%). The stock solution of *Pinus densiflora* needle supercritical fluid extract (PDN-SCFE) was prepared using dimethyl sulfoxide (DMSO) at a concentration of 50 mg/mL, sterilized twice using a DMSO-Safe Acrodisc® Syringe Filter, 0.2 μm pore size (Pall Corporation, NY, USA), and stored at −20 °C for further use.

### Cell culture

RAW 264.7 macrophage cells were cultured in DMEM supplemented with 10% *v*/v HI-FBS, 100 U/mL penicillin, 100 μg/mL streptomycin sulphate and 0.25 μg/mL amphotericin B. Cells were grown at 37 °C under a humidified atmosphere of 5% CO_2_.

### Cell viability assay

The effect of PDN-SCFE on macrophage cell viability was determined using the MTT assay. Briefly, cells were seeded into 96-well plates at a density of 1 × 10^6^ cells/mL and then exposed to various concentrations of PDN-SCFE for 24 h. The concentration of vehicle (DMSO) in all groups including control was 0.08%. Subsequently, 20 μL of MTT stock solution (5 mg/mL) was added to each well. After further incubation for 4 h at 37 °C and 5% CO_2,_ supernatant was removed and formazan crystals were dissolved in DMSO. The absorbance of the solution was measured at 570 nm on a micro-plate reader.

### Nitric oxide (NO) and cytokines assay

RAW 264.7 cells (1 × 10^6^ cells/mL) were cultured in 6-well plates and incubated for 12–16 h. After treatment with PDN-SCFE for 2 h, followed by LPS (1 μg/mL) for 18 h, cell culture supernatants were collected and centrifuged at 800×g and 4 °C, for 5 min. Then, an equal volume of cell-free supernatant was mixed with Griess reagent and incubated for 10 min, at room temperature. The absorbance of the reaction mixture was measured at 540 nm, and NO level was determined by using sodium nitrite as the standard. The levels of cytokines TNFα, IL-6, and IL-1β in supernatants were measured using ELISA kits, according to the manufacturer’s instructions [18].

### Western blot analysis

Cells (1 × 10^6^ cells/mL) were seeded into 60-mm dishes and allowed to adhere overnight. After treatment with PDN-SCFE and/or LPS, cells were washed twice with ice-cold phosphate buffer saline (PBS), and whole cell lysates were prepared using Cellytic M buffer (Sigma) supplemented with protease and phosphatase inhibitors (Roche). The amount of total protein in the lysates was determined using the Bio-Rad protein assay reagent. Lysates containing an equal amount of proteins were electrophoresed using a 8–10% SDS-PAGE gels, and proteins were blotted onto nitrocellulose membrane. These membranes were blocked with either 5% *w*/*v* non-fat milk or 5% *w*/*v* BSA for 1 h, and the target proteins were conjugated using specific primary antibodies, overnight at 4 °C. Further, these processed membranes were washed with Tris-buffered saline containing 0.1% Tween 20 (TBST) and incubated with the suitable HRP-conjugated secondary antibody for 1 h, at room temperature. Afterwards, ECL reagent was used to develop signals, and the results were recorded with a LI-COR image processing system.

### Immunoblotting of NFκB using automated Wes-ProteinSimple system

Cells were pre-treated with PDN-SCFE for 2 h and then stimulated with LPS for 30 min. To prepare nuclear fractions, cells were pelleted and mixed with hypotonic buffer (10 mmol/L HEPES at pH 7.9, 10 mmol/L KCl, 1.5 mmol/L MgCl_2_, 0.1 mmol/L EDTA, 0.5 mmol/L dithiothreitol, and protease and phosphatase inhibitors). After 10 min of incubation on ice, the cell suspensions were supplemented with NP-40 (non-ionic detergent) to a final concentration of 0.1%. The resulting cell suspension was agitated for 15 s, and centrifuged for 1 min, at 15,000×g and 4 °C. The pellets were collected, washed twice with hypotonic buffer without detergent, and lysed in nuclear lysis buffer (20 mmol/L HEPES at pH 7.9, 420 mmol/L NaCl, 1.5 mmol/L MgCl_2_, 1.0 mmol/L EDTA, 1.0 mmol/L dithiothreitol, and protease and phosphatase inhibitors), for 15 min, on ice. Nuclear lysates were centrifuged for 15 min at 4 °C and 15,000×g. The supernatant (nuclear fraction) was collected. The total protein concentrations of the fractions were determined using a BCA protein assay kit (Sigma). The levels of NFκB were determined using automated Wes-ProteinSimple instrument according to the manufacturer’s protocol. In brief, 4 parts lysate (equal amount of protein) were mixed with 1 part fluorescent master mix containing molecular weight markers (ProteinSimple) and dithiothreitol (final concentration 40 mM) and then heated at 95 °C for 5 min. The samples, biotinylated protein ladder (ProteinSimple), and its respective secondary conjugate (ProteinSimple), blocking reagent (ProteinSimple), primary antibodies (NFκB or Lamin B), HRP-conjugated secondary antibody (ProteinSimple), chemiluminescent substrate (ProteinSimple), and wash buffer (ProteinSimple), were loaded into the prefilled microplates containing the separation and stacking matrices (ProteinSimple). After plate loading, the separation electrophoresis and immunodetection steps took place in the capillary system and were fully automated [19].

### Fluorescent microscopy

RAW 264.7 macrophage cells were grown on poly-L-lysine coated glass coverslips placed in 6-well plates. After treatment with PDN-SCFE and/or LPS, cells were fixed using 4% *w*/*v* buffered formalin for 20 min, and then permeabilized with 0.2% *v*/v Triton X-100 (non-ionic detergent) for 10 min, at room temperature. Non-specific regions were blocked with 3% *w*/*v* BSA for 30 min, at room temperature. To assess the cellular localization of NFκB, cells were incubated with rabbit anti-NFκB antibody (1:500 in PBS with 1% BSA) overnight, at 4 °C. After washing with Phosphate-buffered saline containing 0.1% Tween-20 (PBST), cells were incubated with goat anti-rabbit Alexa Fluor® 488 secondary antibody for 60 min, in the dark. After a final wash with PBST, samples were mounted in anti-fade reagent, and signals were captured using a Nikon fluorescence microscope.

### Quantitative real time-polymerase chain reaction (qRT-PCR)

Cells (1 × 10^6^ cells/mL) were seeded into 60-mm dishes, and allowed to adhere overnight. After desired treatment, total RNA of cells was extracted using Trizol reagent, according to the Abcam RNA isolation protocol. cDNA was synthesized using an iScriptTM cDNA Synthesis kit (Bio-rad), according to the instructions given by the manufacturer. qRT-PCR was performed using specific primers in the customer iQTM SYBR Green Super mix (Bio-rad) with the following conditions: 95 °C for 3 min, 40 cycles of 95 °C for 15 s, and 55–60 °C for 30 s. Subsequently, a melt curve analysis was performed to confirm the absence of non-specific signals. The expression levels of all assayed mRNAs were normalized with the mean Ct value of a control gene, β-actin [18]. The PCR primers used in this study were: iNOS, CAT GCT ACT GGA GGT GGG TG (forward), CAT TGA TCT CCG TGA CAG CC (reverse); TNFα, AGC ACA GAA AGC ATG ATC CG (forward), CTG ATG AGA GGG AGG CCA TT (reverse); IL-6, GAG GAT ACC ACT CCC AAC AGA CC (forward), AAG TGC ATC ATC GTT GTT CAT ACA (reverse); IL-1β, TGC AGA GTT CCC CAA CTG GTA CAT C (forward), GTG CTG CCT AAT GTC CCC TTG AAT C (reverse); and β-actin, ATC ACT ATT GGC AAC GAG CG (forward), TCA GCA ATG CCT GGG TAC AT (reverse).

### Statistical analysis

All experiments were repeated three independent times with similar trends, and data were analysed using the Prism 7.02 software (GraphPad, San Diego, CA). The obtained, mean ± SD values are presented in the Figs. A One-way Analysis of Variance and Dunnet or Tukey were used to analyse the significant difference between the experimental groups. *P-*values of < 0.05, 0.01 or 0.001 were considered statistically significant.

## Results

### Effect of PDN-SCFE on RAW 264.7 macrophage cell viability

**Fig. 1:**
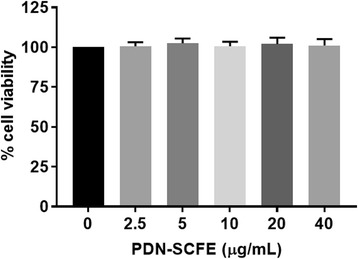
Effect of PDN-SCFE on RAW 264.7 murine macrophage cell viability. Cells were treated with indicated concentrations of PDN-SCFE for 24 h. Then, cell viability was determined by MTT assay as described in methods. The viability of cells in the absence of extract was taken as 100% (Control). The results are expressed by percentage of surviving cells over control group. Data represent mean ± SD of three separate experiments

The MTT assay was performed to determine a non-toxic concentration of PDN-SCFE to use in evaluating its anti-inflammatory potential in RAW 264.7 murine macrophages. PDN-SCFE, up to a maximum concentration of 40 μg/mL, did not induce significant toxicity in RAW 264.7 macrophage cells when compared to vehicle-treated control cells (Fig. 1). However, we used only 50% of this concentration for all subsequent experimental assays.

### PDN-SCFE inhibits LPS-induced NO production in RAW 264.7 cells

**Fig. 2:**
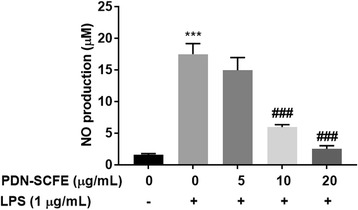
Effect of PDN-SCFE on LPS-induced nitric oxide (NO) production in RAW 264.7 cells. Cells were pre-treated with indicated concentrations of PDN-SCFE for 2 h, and incubated with LPS for another 18 h. The amount of NO in cell-free supernatant was quantified by the Griess method. Data represent mean ± SD of three separate experiments. ^***^ *P* < 0.001 vs control group; ^#^ *P* < 0.05 and ^###^ *P* < 0.001 vs LPS-stimulated group

As shown in Fig. 2, when compared to the vehicle-treated control, LPS noticeably (11.4 fold) increased the production of NO. However, PDN-SCFE dose-dependently (14.3% at 5 μg/mL, 66% at 10 μg/mL and 85.7% at 20 μg/mL) reduced the NO levels in LPS-challenged murine macrophages.

### PDN-SCFE inhibits LPS-induced iNOS expression in RAW 264.7 cells

**Fig. 3:**
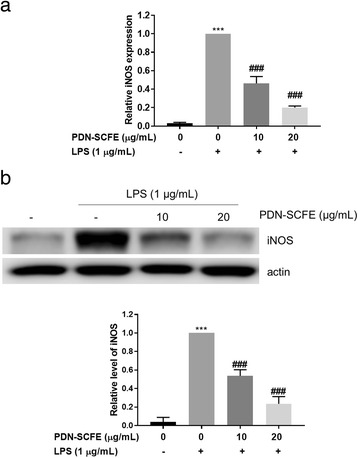
Effect of PDN-SCFE on LPS-induced iNOS (**a**) mRNA and (**b**) protein expression in RAW 264.7 cells. Cells were pre-treated with indicated concentrations of PDN-SCFE for 2 h, followed by LPS for 6 h (mRNA expression) or 18 h (protein expression). The protein and mRNA levels of iNOS were determined by Immuno-blot and qRT-PCR methods, respectively. A representative blot from three separate experiments is shown here. The graphical figures represent the change in protein or mRNA expression normalized to actin. Data represent mean ± SD of three separate experiments. ^***^ *P* < 0.001 vs control group; ^###^ *P* < 0.001 vs LPS-stimulated group

To further substantiate the inhibitory role of PDN-SCFE on NO production in macrophages, we assessed the effect of PDN-SCFE on LPS-induced corresponding enzyme iNOS expression levels. LPS treatment led to an increase in the expression of iNOS mRNA (33 fold) and protein (25.6 fold) levels. PDN-SCFE at 20 μg/mL concentration inhibited 79.9% of iNOS mRNA and 76.4% iNOS protein expression (Fig. 3).

### PDN-SCFE attenuates LPS-induced production of pro-inflammatory cytokines in RAW 264.7 cells

**Fig. 4:**
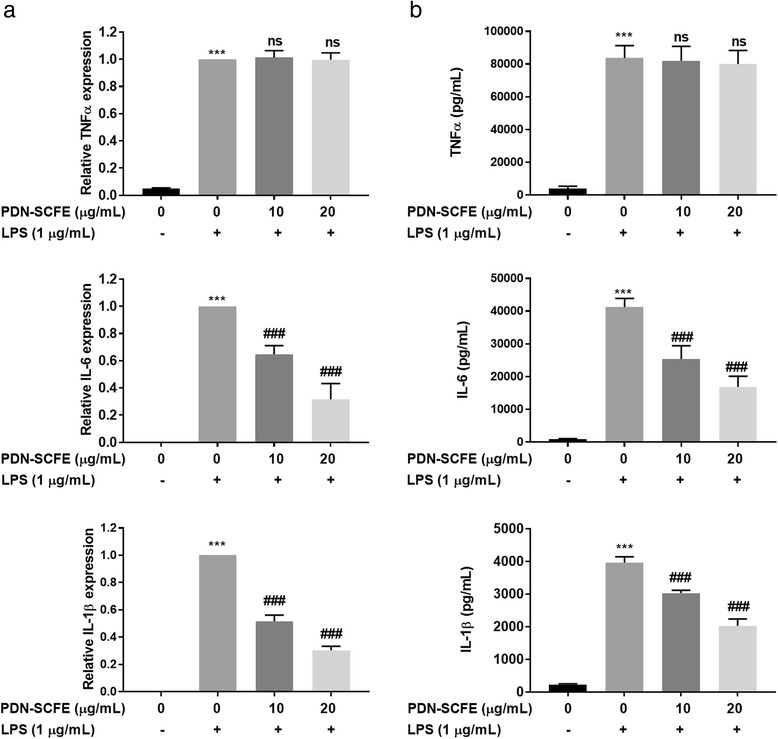
Effect of PDN-SCFE on LPS-induced cytokine (**a**) mRNA and (**b**) protein expression in RAW 264.7 cells. The cells were pre-treated with indicated concentrations of PDN-SCFE for 2 h, followed by LPS for 6 h (a, mRNA expression) or 18 h (b, protein expression) The cell-free supernatant was subjected to quantify TNFα, IL-6 and IL-1β levels, using ELISA kits, according to manufacturer’s protocol. Total RNA was extracted with Trizol reagent and subjected to qRT-PCR according to the manufacturer’s instructions. The graphical figures represent the change in mRNA expression normalized to actin. Data represent mean ± SD from three separate experiments. ^***^ *P* < 0.001 vs control group; ^###^ *P* < 0.001 vs LPS-stimulated group; ns-non significant vs LPS-stimulated group

Next, we tested the effect of PDN-SCFE on LPS-induced production of the major cytokines, TNFα, IL-6 and IL-1β in RAW 264.7 macrophage cells. These cytokines levels were assayed by ELISA and qRT-PCR methods. As shown in Fig. 4, challenging the cells with LPS lead to increased production of TNFα (mRNA, 20.7 fold and protein, 21.2 fold), IL-6 (mRNA, 353 fold and protein 35 fold) and IL-1β (mRNA, 150 fold and protein, 17.9 fold) in RAW 264.7 cells. The increased levels of IL-6 and IL-1β cytokines were noticeably decreased both at mRNA and protein levels in PDN-SCFE-treated cells. Treatment with PDN-SCF at 10 μg/mL decreased levels to 35.3% (IL-6 mRNA), 38.5% (IL-6 protein), 48.3% (IL-1β mRNA) and 23.5% (IL-1β protein) whereas at 20 μg/mL, the levels were decreased to 68.3% (IL-6 mRNA), 59.1% (IL-6 protein), 69.7% (IL-1β mRNA) and 48.8% (IL-1β protein). However, PDN-SCFE did not alter TNFα expression, both at mRNA and protein levels.

### The anti-inflammatory mechanism of PDN-SCFE

**Fig. 5:**
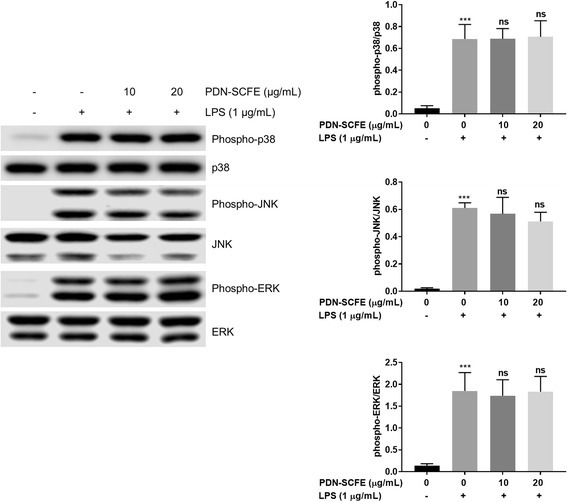
Effect of PDN-SCFE on the MAPK pathway in RAW 264.7 cells. Cells were pre-treated with PDN-SCFE for 2 h, followed by LPS stimulation for 30 min. Then, whole cell lysates were prepared and used to analyze the levels of phosphorylated and total p38, JNK and ERK by an immune-blotting assay. Blots are representative of three independent experiments. The graphical figures represent the relative change in the ratio of phosphorylated to total protein levels. ^***^ *P* < 0.001 vs control group; ns-non significant vs LPS-stimulated group

**Fig. 6:**
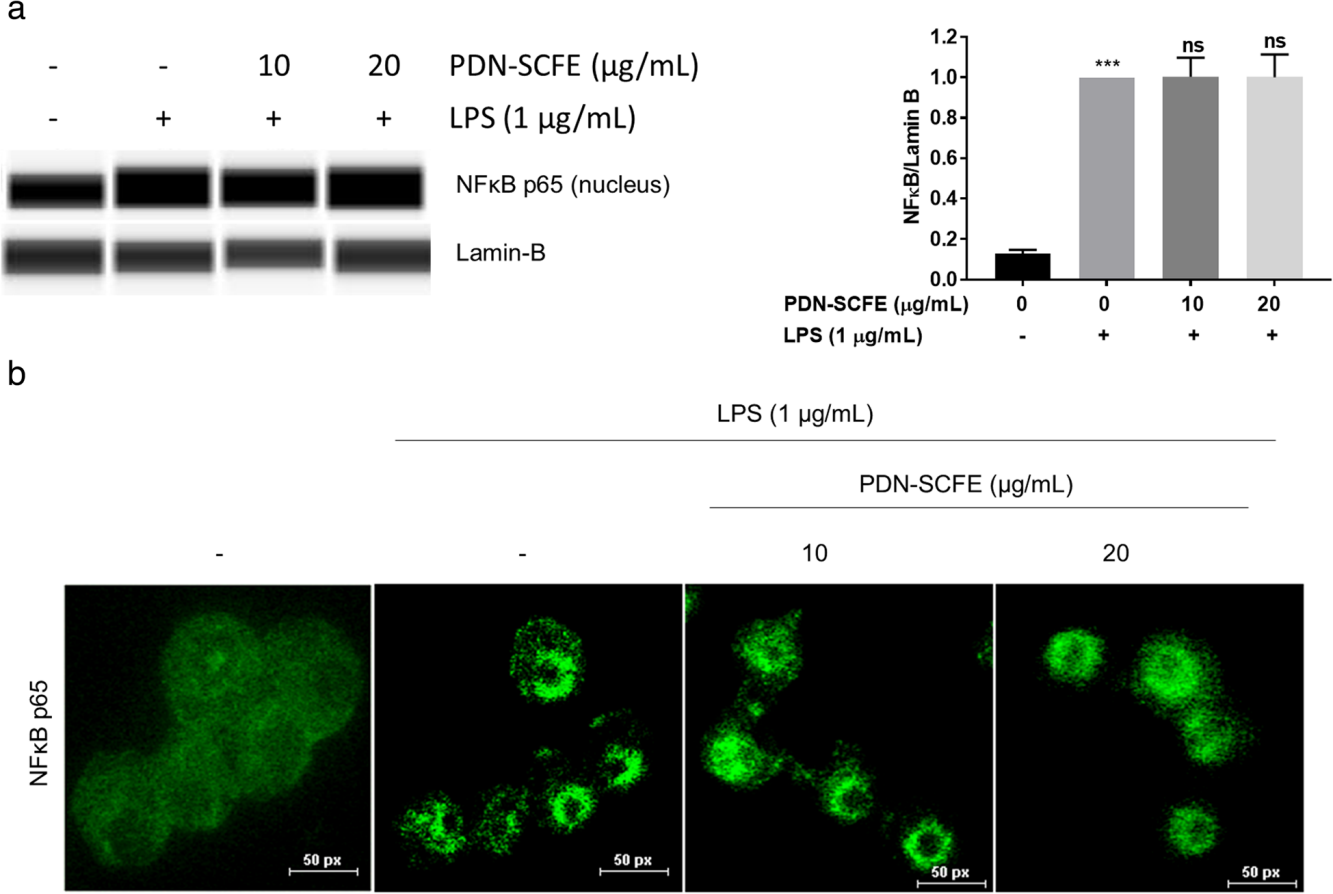
Effect of PDN-SCFE on the NFκB pathway in RAW 264.7 cells. Cells were pre-treated with PDN-SCFE for 2 h, followed by LPS stimulation for 30 min. **a** nuclear fractions were prepared and used to analyze the levels of NFκB p65 by an immune-blotting assay using the automated Wes-proteinsimple system. Blots are representative of three independent experiments. The graphical figures represent the relative change in NFκB p65 protein levels normalized to Lamin B. ^***^ *P* < 0.001 vs control group; ns-non significant vs LPS-stimulated group. **b** the translocation of NFκB p65 to the nucleus was assessed by immunofluorescence staining. Representative microscopic images are shown here

In the present study, we observed that PDN-SCFE has no inhibitory effect on LPS-induced activation of MAPKs such as P38, JNK and ERK (Fig. 5), as well as nuclear translocation of NFκB in RAW 264.7 cells (Fig. 6).

**Fig. 7:**
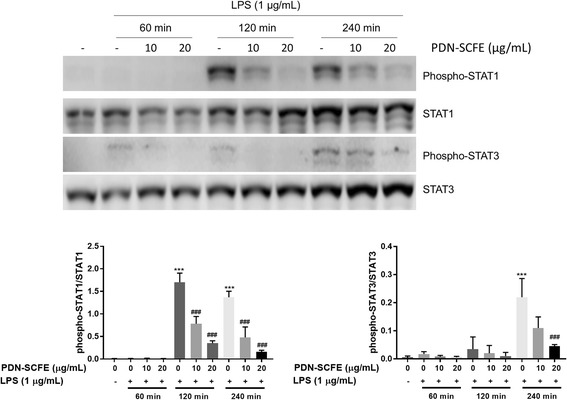
Effect of PDN-SCFE on LPS-induced activation of the JAK-STAT pathway in RAW 264.7 cells. Cells were pre-treated with PDN-SCFE for 2 h, followed by LPS stimulation for 60–240 min. Then, whole cell lystaes were prepared and used to analyze the levels of phosphorylated and non-phosphorylated form of STAT1 and STAT3 by an immune-blotting assay. Blots are representative of three independent experiments. The graphical figures represent the relative change in the ratio of phosphorylated to total protein levels. ^***^ *P* < 0.001 vs control group; ^###^ *P* < 0.001 vs LPS-stimulated group; ^##^ *P* < 0.01 vs LPS-stimulated group; ns-non significant vs LPS-stimulated group

Since the STAT signaling pathway is considered to be an important regulatory pathway for iNOS, IL-6, and IL-1β expression in macrophages [20], and as this study demonstrated decreased levels of these inflammatory molecules, we intended to study the effect of PDN-SCFE on the STAT pathway. As shown in Fig. 7, phosphorylation of STAT1 and STAT3 in LPS-challenged cells was remarkably increased, time-dependently. However, PDN-SCFE showed significant inhibitory effect on LPS-induced phosphorylation of these signaling molecules. From these results, it seems reasonable to say that PDN-SCFE may decrease the production of NO, IL-6, and IL-1β through inhibition of STAT proteins.

## Discussion

The supercritical fluid extraction method has emerged as an alternative to the traditional process where toxic hydrocarbon or chlorinated organic solvents are used for extraction. In this method, non-toxic carbon dioxide alone or in combination with ethanol is most commonly used. This method has several advantages, which include the simple recovery of components, the quick process that usually does not last more than one hour, the superior quality, higher yield and absence of residual solvent left in extracts, and the inexpensive and environment-friendly process [21]. Hence, in the present study, we adopted this method for preparation of *Pinus densiflora* needle extract.

*Pinus densiflora* is identified as a common pine species found in Korea. Traditionally, it is believed that the needle extract of this plant can be used for treatment of rheumatitis, hemorrhage and cancer. Further, existing scientific studies have clearly demonstrated that *Pinus densiflora* needle extracts possess various pharmacological properties including antioxidant, anti-mutagenic, antitumor and antibacterial effects [22–24]. The present study revealed that *Pinus densiflora* needle supercritical fluid extract inhibits the production of inflammatory molecules, NO, IL-6 and IL-1β in LPS-stimulated murine RAW 264.7 macrophage cells.

In general, the systemic immune system tightly regulates the production of inflammatory mediators from activated macrophages. However, failure of this regulatory process in various disease conditions causes inflammation that can lead to severe tissue damage [25]. Therefore, novel therapeutic intervention is necessary to regulate the increased production of inflammatory molecules in macrophages. NO is known to play vital biological roles such as vasodilation, platelet aggregation, smooth muscle cell proliferation and migration, inflammation and apoptotic cell death [26–29]. Generally, NO is protective at physiological level. However, an abnormal level of this signaling molecule is potentially toxic to tissues [30]. Thus, down-regulating the production of NO is needed for controlling the pathogenesis of disease complications. LPS induces an inflammatory response in macrophages by increasing the expression of iNOS, the enzyme that synthesizes NO [31]. In addition to NO, LPS-induced inflammatory responses are coordinated by the increased production of various pro-inflammatory cytokines such as TNFα, IL-6 and IL-1β [32]. Our experimental results show that PDN-SCFE had a significant inhibitory effect on NO production and the corresponding enzyme iNOS expression, as well as IL-6 and IL-1β, but not TNFα expression in LPS-challenged RAW 264.7 macrophages.

LPS-induced increased expression of iNOS, TNFα, IL-6 and IL-1β in macrophages are under the control of NFκB, MAPKs, JAK-STAT and also by various inflammatory signaling pathways [33–35]. NFκB, is largely sequestered and inactivated by the inhibitory protein, IκB, in the cytoplasm. LPS activates and releases the free active NFκB by inducing degradation of IκB via the ubiquitin proteasome pathway. Consequently, this allows the translocation of NFκB into nucleus, where it binds to DNA and targets the expression of various inflammatory molecules [36]. In our study, the automated western and immunofluorescence results reveal that PDN-SCFE did not affect the LPS-induced increased nuclear levels of NFκB in murine macrophages. Existing reports have clearly demonstrated that the three major subfamilies of MAPKs such as P38, JNK and ERK are potential targets for anti-inflammatory therapeutics owing to their involvement in the regulation of the expression of pro-inflammatory mediators, both at transcriptional and translational levels [37, 38]. Unfortunately, PDN-SCFE did not hamper the LPS-induced activation of these major MAPKs in murine macrophages.

The JAK-STAT pathway comprises the principal signaling target for a wide range of cytokines and growth factors as well as lipopolysaccharides [39]. At first, LPS stimulates the phosphorylation and activation of JAK proteins which subsequently activates its major substrates, STAT proteins in macrophages [11]. Once activated, STATs can enter the nucleus where they bind to specific regulatory sequences in DNA either to activate or repress the expression of various target genes including pro-inflammatory genes [40]. Studies have reported that among the seven members of STAT proteins, STAT1 is crucial for iNOS, and STAT3 for IL-6 and IL-1β expressions in response to LPS, in macrophages [41, 42]. Further, Guo et al. (2014) have demonstrated that cyclovirobuxinum D, an alkaloid from the Chinese medicinal plant *Buxus microphylla* reduces iNOS, IL-6 and IL-1β via inhibiting STAT1 and STAT3 activation in LPS-stimulated RAW 264.7 cells [20]. Since PDN-SCFE significantly reduced the LPS-induced iNOS, IL-6 and IL-1β, we investigated the effect of PDN-SCFE on STAT1 and STAT3 activation. Our results provide evidence that PDN-SCFE has significant suppressive effects on the activation of STAT1 and STAT3 in LPS-challenged RAW 264.7 macrophages.

## Conclusion

The results of this study revealed that *Pinus densiflora* needle supercritical fluid extract has a significant inhibitory effect on LPS-induced expression of pro-inflammatory mediators, iNOS, IL-6 and IL-1β in RAW264.7 macrophage cells. Further, the extract-induced, inhibitory effect on the phosphorylation-mediated activation of STAT1 and STAT3 may be responsible for the decreased expression of these pro-inflammatory mediators. Further studies are warranted to identify the active principles in this pine needle supercritical fluid extract responsible for its anti-inflammatory activity in murine macrophages.

## Abbreviations

BCA: Bicinchoninic acid
BSA: Bovine serum albumin
cDNA: complementary deoxyribonucleic acid
DMEM: Dulbecco’s modified Eagle’s medium
DMSO: Dimethyl sulfoxide
*E. coli*: *Escherichia coli*
ECL: Enhanced chemiluminescence
EDTA: Ethylenediaminetetraacetic acid
ELISA: Enzyme-linked immunosorbent assay
ERK: Extracellular signal–regulated kinase
HEPES: 4-(2-Hydroxyethyl)piperazine-1-ethanesulfonic acid, N-(2-Hydroxyethyl)piperazine-N′-(2-ethanesulfonic acid)
HI-FBS: heat-inactivated fetal bovine serum
HRP: Horseradish peroxidase
IL-1β: Interleukin-1β
IL-6: Interleukin-6
iNOS: Inducible nitric oxide synthase
IκB: Inhibitory kappa B protein
JAK-STAT: Janus kinase-signal transducers and activators of transcription
JNK: c-Jun N-terminal kinase
KCl: Potassium chloride
KCLB: Korean Cell Line Bank
LPS: lipopolysaccharide
MAPKs: Mitogen-activated protein kinases
MgCl_2_: Magnesium chloride
mRNA: messenger RNA
MTT: (3-(4,5-Dimethylthiazol-2-yl)-2,5-Diphenyltetrazolium Bromide)
NaCl: Sodium chloride
NFκB: Nuclear transcription factor kappa B
NO: Nitric oxide
P38: P38 Mitogen-activated protein kinase
PBST: Phosphate-buffered saline with detergent Tween 20
PDN-SCFE: *Pinus densiflora* needle supercritical fluid extract
PGE2: Prostaglandin E2
qRT-PCR: Quantitative real-time polymerase chain reaction
RNA: Ribonucleic acid
SDS-PAGE: Sodium dodecyl sulfate-polyacrylamide electrophoresis
STAT1: Signal transducers and activators of transcription factor 1
STAT3: Signal transducers and activators of transcription factor 3
TBST: Tris-buffered saline with detergent Tween 20
TLR4: Toll-like receptor 4
TNFα: Tumor necrosis factor α

## References

[1] Rock KL, Kono H (2008). The inflammatory response to cell death. Annu Rev Pathol.

[2] Valledor AF, Comalada M, Santamaría-Babi LF, Lloberas J, Celada A (2010). Macrophage proinflammatory activation and deactivation: a question of balance. Adv Immunol.

[3] Wong SH, Lord JM (2004). Factors underlying chronic inflammation in rheumatoid arthritis. Arch Immunol Ther Exp.

[4] Libby P (2006). Inflammation and cardiovascular disease mechanisms. Am J Clin Nutr.

[5] Porta C, Larghi P, Rimoldi M, Totaro MG, Allavena P, Mantovani A (2009). Cellular and molecular pathways linking inflammation and cancer. Immunobiology.

[6] Wee YV (2010). Inflammation in neurological disorders: a help or a hindrance?. Neuroscientist.

[7] Esser N, Legrand-Poels S, Piette J, Scheen AJ, Paquot N (2014). Inflammation as a link between obesity, metabolic syndrome and type 2 diabetes. Diabetes Res Clin Pract.

[8] Italiani P, Boraschi D (2015). New Insights Into Tissue Macrophages: From Their Origin to the Development of Memory. Immune Netw.

[9] Arango Duque G, Descoteaux A (2014). Macrophage cytokines: involvement in immunity and infectious diseases. Front Immunol.

[10] Fujihara M, Muroi M, Tanamoto K, Suzuki T, Azuma H, Ikeda H (2003). Molecular mechanisms of macrophage activation and deactivation by lipopolysaccharide: roles of the receptor complex. Pharmacol Ther.

[11] Okugawa S, Ota Y, Kitazawa T, Nakayama K, Yanagimoto S, Tsukada K (2003). Janus kinase 2 is involved in lipopolysaccharide-induced activation of macrophages. Am J Physiol Cell Physiol.

[12] Kou X, Qi S, Dai W, Luo L, Yin Z (2011). Arctigenin inhibits lipopolysaccharide-induced iNOS expression in RAW264.7 cells through suppressing JAK-STAT signal pathway. Int Immunopharmacol.

[13] Jeon JR, Kim JY (2006). Effects of pine needle extract on differentiation of 3T3-L1 preadipocytes and obesity in high-fat diet fed rats. Biol Pharm Bull.

[14] Süntar I, Tumen I, Ustün O, Keleş H, Akkol EK (2012). Appraisal on the wound healing and anti-inflammatory activities of the essential oils obtained from the cones and needles of Pinus species by in vivo and in vitro experimental models. J Ethnopharmacol.

[15] Wu Y, Bai J, Zhong K, Huang Y, Qi H, Jiang Y, et al. Antibacterial Activity and Membrane-Disruptive Mechanism of 3-p-trans-Coumaroyl-2-hydroxyquinic Acid, a Novel Phenolic Compound from Pine Needles of *Cedrus deodara*, against *Staphylococcus aureus*. Molecules. 2016;21(8):1084. 10.3390/molecules21081084 PMC6273998 27548123

[16] Shi X, Liu D, Zhang J, Hu P, Shen W, Fan B (2016). Extraction and purification of total flavonoids from pine needles of Cedrus deodara contribute to anti-tumor in vitro. BMC Complement Altern Med.

[17] Choi H-S, Hang D, Cho S-J, Kang S-C, Sohn E-S (2006). Immunomodulatory Activity of Pine Needle (Pinus densiflora) Extracts in Macrophages. J Food Sci Nutr.

[18] Li RJ, Gao CY, Guo C, Zhou MM, Luo J, Kong LY (2017). The Anti-inflammatory Activities of Two Major Withanolides from Physalis minima Via Acting on NF-κB, STAT3, and HO-1 in LPS-Stimulated RAW264.7 Cells. Inflammation.

[19] Thamizhiniyan V, Young-Woong C, Young-Kyoon K (2015). The cytotoxic nature of Acanthopanax sessiliflorus stem bark extracts in human breast cancer cells. Saudi J Biol Sci.

[20] Guo D, Li JR, Wang Y, Lei LS, Yu CL, Chen NN (2014). Cyclovirobuxinum D suppresses lipopolysaccharide-induced inflammatory responses in murine macrophages in vitro by blocking JAK-STAT signaling pathway. Acta Pharmacol Sin.

[21] Durante M, Lenucci MS, Mita G (2014). Supercritical carbon dioxide extraction of carotenoids from pumpkin (Cucurbita spp.): a review. Int J Mol Sci.

[22] Jung MJ, Chung HY, Choi JH, Choi JS (2003). Antioxidant principles from the needles of red pine, Pinus densi fl ora. Phytother Res.

[23] Hong EJ, Na KJ, Choi IG, Choi KC, Jeung EB (2004). Antibacterial and antifungal effects of essential oils from coniferous trees. Biol Pharm Bull.

[24] Kwak CS, Moon SC, Lee MS (2006). Antioxidant, antimutagenic, and antitumor effects of pine needles (Pinus densiflora). Nutr Cancer.

[25] Laskin DL, Sunil VR, Gardner CR, Laskin JD (2011). Macrophages and tissue injury: agents of defense or destruction?. Annu Rev Pharmacol Toxicol.

[26] Ignarro LJ (1996). Physiology and pathophysiology of nitric oxide. Kidney Int Suppl.

[27] Guzik TJ, Korbut R, Adamek-Guzik T (2003). Nitric oxide and superoxide in inflammation and immune regulation. J Physiol Pharmacol.

[28] Li CQ, Wogan GN (2005). Nitric oxide as a modulator of apoptosis. Cancer Lett.

[29] Tsihlis ND, Oustwani CS, Vavra AK, Jiang Q, Keefer LK, Kibbe MR (2011). Nitric oxide inhibits vascular smooth muscle cell proliferation and neointimal hyperplasia by increasing the ubiquitination and degradation of UbcH10. Cell Biochem Biophys.

[30] Guix FX, Uribesalgo I, Coma M, Muñoz FJ (2005). The physiology and pathophysiology of nitric oxide in the brain. Prog Neurobiol.

[31] Jacobs AT, Ignarro LJ (2001). Lipopolysaccharide-induced expression of interferon-beta mediates the timing of inducible nitric-oxide synthase induction in RAW 264.7 macrophages. J Biol Chem.

[32] Schulte W, Bernhagen J, Bucala R (2013). Cytokines in sepsis: potent immunoregulators and potential therapeutic targets--an updated view. Mediat Inflamm.

[33] Zhai XT, Zhang ZY, Jiang CH, Chen JQ, Ye JQ, Jia XB (2016). Nauclea officinalis inhibits inflammation in LPS-mediated RAW 264.7 macrophages by suppressing the NF-κB signaling pathway. J Ethnopharmacol.

[34] Hwang YJ, Lee SJ, Park JY, Chun W, Nam SJ, Park JM (2016). Apocynin Suppresses Lipopolysaccharide-Induced Inflammatory Responses Through the Inhibition of MAP Kinase Signaling Pathway in RAW264.7 Cells. Drug Dev Res.

[35] Pan X, Cao X, Li N, Xu Y, Wu Q, Bai J (2014). Forsythin inhibits lipopolysaccharide-induced inflammation by suppressing JAK-STAT and p38 MAPK signalings and ROS production. Inflamm Res.

[36] Tak PP, Firestein GS (2001). NF-kappaB: a key role in inflammatory diseases. J Clin Invest.

[37] Roux PP, Blenis J (2004). ERK and p38 MAPK-activated protein kinases: a family of protein kinases with diverse biological functions. Microbiol Mol Biol Rev.

[38] Kaminska B (2005). MAPK signalling pathways as molecular targets for anti-inflammatory therapy--from molecular mechanisms to therapeutic benefits. Biochim Biophys Acta.

[39] Jatiani SS, Baker SJ, Silverman LR, Reddy EP (2010). Jak/STAT pathways in cytokine signaling and myeloproliferative disorders: approaches for targeted therapies. Genes Cancer.

[40] Rawlings JS, Rosler KM, Harrison DA (2004). The JAK/STAT signaling pathway. J Cell Sci.

[41] Ohmori Y, Hamilton TA (2001). Requirement for STAT1 in LPS-induced gene expression in macrophages. J Leukoc Biol.

[42] Samavati L, Rastogi R, Du W, Hüttemann M, Fite A, Franchi L (2009). STAT3 tyrosine phosphorylation is critical for interleukin 1 beta and interleukin-6 production in response to lipopolysaccharide and live bacteria. Mol Immunol.

